# A clinically practical machine learning nomogram for preoperative CLNM prediction in PTMC: tumor-capsule distance and D2–40 evidence

**DOI:** 10.3389/fonc.2026.1806758

**Published:** 2026-05-28

**Authors:** Zetao Ling, Xingluo Wang, Li Wu, Bin Chen, Yabing Wang, Rui Sha, Changsheng Yu

**Affiliations:** Department of Breast and Thyroid Surgery, The First Affiliated Hospital of Wannan Medical University, Wuhu, Anhui, China

**Keywords:** central lymph node metastasis, D2-40 staining, machine learning, nomogram, papillary thyroid microcarcinoma, tumor–capsule distance

## Abstract

**Objective:**

This study aimed to develop and validate a machine learning-driven nomogram for preoperative prediction of central lymph node metastasis (CLNM) in papillary thyroid microcarcinoma (PTMC), with a focus on elucidating the role of tumor–capsule distance (TCD) and its underlying biological mechanism via D2–40 immunohistochemical evidence.

**Methods:**

A retrospective cohort of 503 PTMC patients was randomly divided into training, validation, and test sets (6:2:2). Feature selection was performed using XGBoost and interpreted via SHAP analysis. A nomogram was constructed based on logistic regression and validated internally and externally (n = 101). D2–40 staining was used to assess lymphatic vessel density (LVD) in relation to TCD.

**Results:**

Five key predictors were identified: TCD, microcalcifications, age, BMI, and maximum tumor diameter. A TCD < 2 mm was strongly associated with CLNM (p < 0.0001). The XGBoost model achieved an AUC of 0.900 on the test set, while the nomogram showed AUCs of 0.862, 0.836, and 0.875 on the training, validation, and test sets, respectively, with external validation AUC of 0.870. D2–40 staining confirmed significantly higher LVD in the pericapsular region (p < 0.05), supporting the biological plausibility of TCD as a predictor.

**Conclusion:**

The proposed nomogram demonstrates high predictive accuracy, clinical interpretability, and biological grounding. It serves as a practical tool for individualized risk assessment of CLNM in PTMC, potentially guiding precision treatment decisions.

## 1 Introduction

Papillary thyroid microcarcinoma (PTMC) is a special subtype of papillary thyroid carcinoma (PTC) defined as a tumor with a maximum diameter ≤ 1 cm. It accounts for more than 50% of all thyroid malignancies and is generally characterized by the absence of obvious extracapsular invasion, lymph node metastasis (LNM), or distant metastasis (T1aN0M0) (1). The detection rate of PTMC has increased markedly, which is mainly attributed to the widespread application of imaging examination techniques and the introduction of fine-needle aspiration biopsy (2). Most PTMCs progress asymptomatically and present an excellent prognosis, with a 10-year survival rate exceeding 99% (3, 4). This situation has led to ongoing controversy and challenges regarding the optimal management and therapeutic strategies for PTMC.

The American Thyroid Association (ATA) guidelines for thyroid cancer management recommend surgery as the first-line therapeutic strategy for unifocal PTMC with risk factors (5). However, thyroid surgery is associated with high medical costs and a considerable incidence of postoperative complications, including hypoparathyroidism, recurrent laryngeal nerve injury, hemorrhage, infection and cosmetic concerns, which substantially impair patients’ postoperative quality of life (6, 7). Accumulating contemporary studies have indicated that active surveillance or thermal ablation is a safe and feasible alternative for the management of low-risk PTMC (5). Active surveillance requires regular ultrasonographic follow-up of tumor size and cervical lymph nodes, and surgical intervention is only performed when disease progression or evidence of cervical lymph node metastasis is identified (8, 9). Thermal ablation has been proven effective and safe for the treatment of PTMC; nevertheless, its therapeutic efficacy remains uncertain once cervical lymph node metastasis occurs (10). Therefore, accurate preoperative evaluation of cervical lymph node metastatic status is critical for individualized clinical decision-making in PTMC patients.

At present, numerous approaches have been applied to the preoperative prediction of lymph node metastasis in patients with PTMC, including clinical characteristics (e.g., age, tumor size, gender, and multifocality), ultrasonic features (e.g., microcalcification, echogenicity, margin, and aspect ratio), and pathological features (e.g., BRAF and RAS gene mutations) (11, 12). Nevertheless, these methods still have inherent limitations, such as unsatisfactory predictive accuracy, invasiveness, high cost, and lack of unified diagnostic criteria (13, 14). Such deficiencies frequently result in overtreatment or delayed treatment in clinical practice. As a visual predictive tool, nomograms quantify the probability of individual clinical events by integrating multiple prognostic and decision-related variables, and have demonstrated favorable clinical value in the prediction of various malignancies such as gastric cancer and breast cancer. However, the predictive performance and accuracy of existing nomogram models remain to be further improved (15). In recent years, machine learning (ML) has played an increasingly vital role in medical research and clinical decision-making owing to its powerful capability of pattern recognition and outcome prediction. It has been widely applied in cancer detection, diagnostic classification, and the formulation of personalized therapeutic strategies (16–18), enabling the mining of complex latent features from massive clinical data and the construction of predictive models. Although ML models perform well in integrating clinical, ultrasonographic and pathological features to predict tumor invasiveness, their inherent complexity raises concerns regarding model interpretability and generalizability (17). Accordingly, the present study aimed to combine machine learning with nomogram modeling to develop a clinically practical and highly interpretable predictive model, so as to assist clinicians in making optimal decisions for the management and treatment of PTMC.

Furthermore, a rich lymphatic network is distributed around the thyroid capsule (19, 20), and capsular invasion (i.e., tumor-capsule distance = 0) has been confirmed by multiple studies as an independent risk factor for central lymph node metastasis (CLNM) (21, 22). This suggests that the spatial relationship between the tumor and the thyroid capsule may directly affect the risk of lymph node metastasis—when the tumor is adjacent to the capsule, the probability of it contacting lymphatic vessels increases significantly. Based on this anatomical foundation, we propose the hypothesis that Tumor-Capsule Distance (TCD) may serve as a novel imaging marker for predicting CLNM, and there exists a threshold effect between TCD and CLNM. This study will systematically verify the predictive value of TCD and further explore its potential mechanism through histopathological analysis. This innovative design compensates for the insufficient consideration of anatomical spatial factors in existing prediction models.

## 2 Methods

### 2.1 Study subjects

A retrospective cohort study was conducted at the First Affiliated Hospital of Wannan Medical Uiversity. We reviewed the medical records of 503 PTMC patients who underwent thyroidectomy and lymph node dissection between May 2023 and May 2024. According to the “Guidelines for the Diagnosis and Treatment of Thyroid Nodules and Thyroid Cancer in China,” at least unilateral thyroid lobectomy and unilateral central neck lymph node dissection were performed for thyroid cancer patients. For patients with bilateral thyroid cancer, total thyroidectomy and bilateral central neck lymph node dissection were performed. Given the retrospective nature of the study, informed consent was not required.

Inclusion Criteria: 1. Patients with papillary thyroid carcinoma (PTC) confirmed by histopathology, with a maximum tumor diameter of ≤ 10 mm, who underwent thyroid surgery for the first time; 2. Patients who received preoperative ultrasound examination, fine-needle aspiration (FNA) biopsy, and BRAF gene detection, with cytological classification of Bethesda V–VI; 3. Patients with clinically negative cervical lymph nodes (cN0) as evaluated by preoperative neck ultrasound.

Exclusion Criteria: 1. Patients with a history of other malignant tumors or previous neck radiotherapy; 2. Patients with incomplete clinical or pathological data; 3. Patients with obvious extrathyroidal extension (ETE) of the tumor indicated by preoperative ultrasound or imaging examinations.

**Figure 1:**
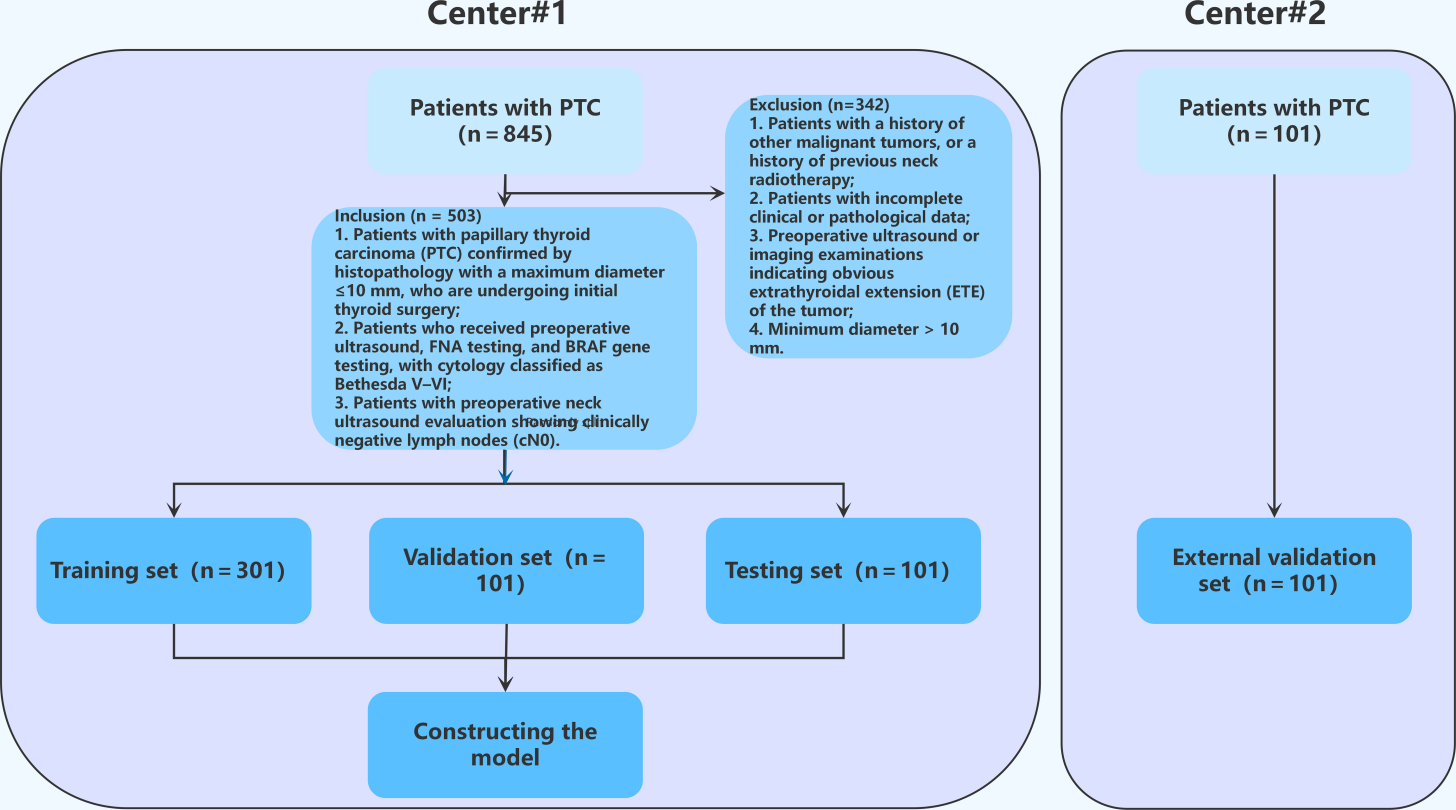
Flowchart of patient enrollment and selection.

A total of 845 patients with papillary thyroid microcarcinoma (PTMC) were initially screened in this study, and 503 patients were finally included in accordance with the aforementioned inclusion and exclusion criteria. The medical records and analytical variables of all included cases were complete without missing data (**Figure 1**).In addition, a total of 101 consecutive PTMC patients who met the same inclusion and exclusion criteria were concurrently enrolled from the Second Affiliated Hospital of Wannan Medical University (Center 2) to form an independent external validation cohort, which was exclusively used to evaluate the final generalization performance of the model.

### 2.2 Data collection and partition

**Figure 2:**
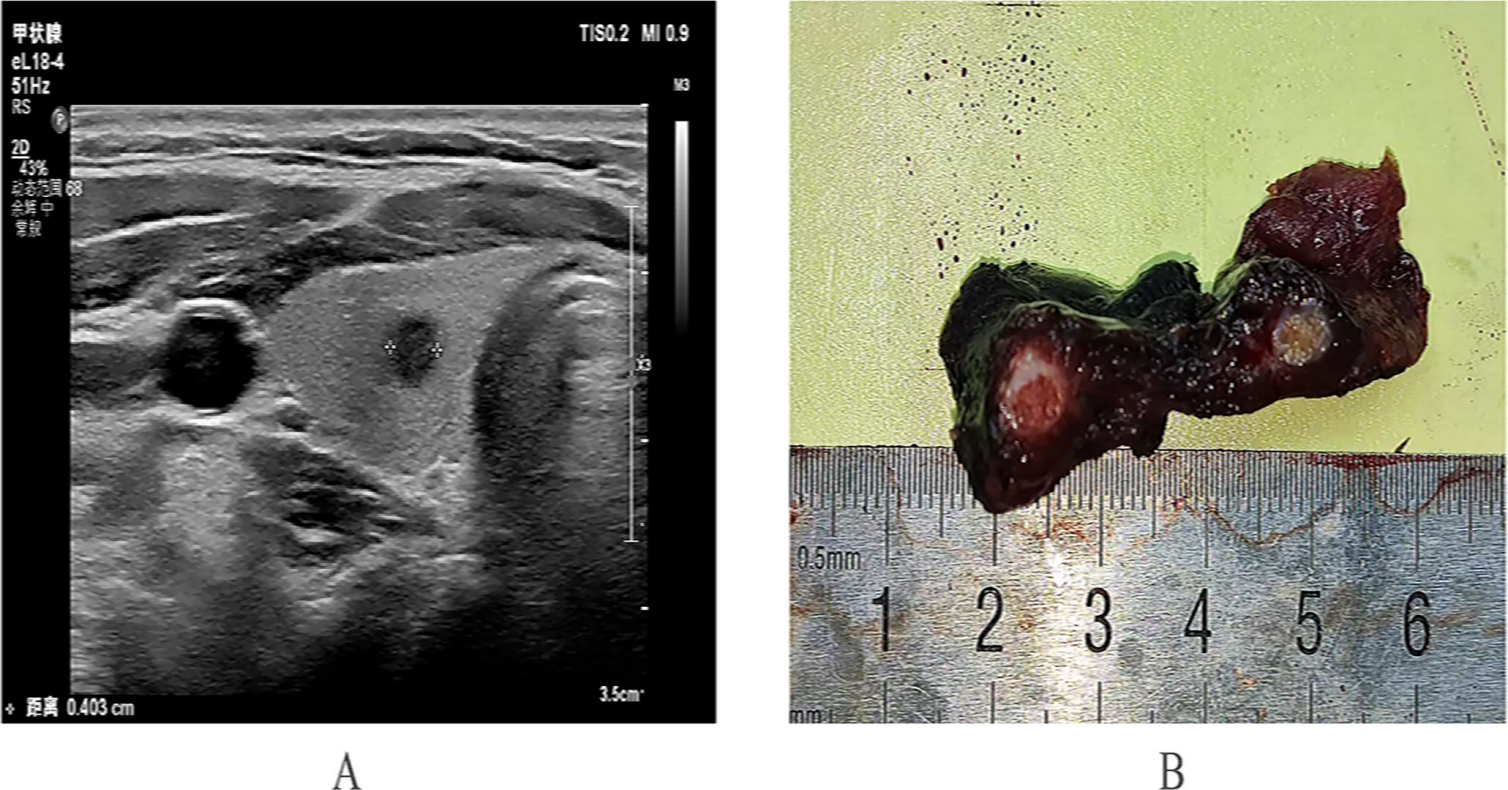
Ultrasound images and pathological specimen. **(A)** Measurement of tumor-to-capsule distance, **(B)** Schematic diagram of a longitudinally sectioned lesion.

The collected clinical data included age, gender, body mass index (BMI, calculated as weight in kilograms divided by the square of height in meters), history of hypertension, history of diabetes, central lymph node metastasis (CLNM), ipsilateral nodular goiter (iNG), and BRAF gene mutation status. The following variables were measured and evaluated based on preoperative ultrasound images: maximum tumor diameter, tumor location, tumor margin, microcalcification, and tumor-capsule distance (TCD). Tumor location was divided into upper 1/3, middle 1/3, lower 1/3, and isthmus according to the long axis of the thyroid gland. Multifocality and Hashimoto’s thyroiditis (HT) were confirmed by postoperative histopathological examination; multifocality was defined as the presence of two or more PTMC lesions in the thyroid gland. Tumor size was defined as the longest three-dimensional diameter. Tumor-capsule distance was defined as the shortest distance from the tumor boundary to the thyroid capsule on the transverse view, and all measurements were independently performed by a senior radiologist who was blinded to the clinical information (**Figure 2A**). Regarding ultrasound features, tumor margins were classified as smooth or irregular. The diagnosis of CLNM was confirmed by pathological examination of the resected lymph nodes. Clinically negative cervical lymph nodes (cN0) were defined as the absence of suspicious cervical lymph nodes, or lymph nodes without any of the following suspicious ultrasound features: microcalcification, cystic degeneration, peripheral or diffuse hyperechogenicity, round shape (aspect ratio < 2), disappearance of the lymph node hilum, or disordered blood flow signals (13).

D2–40 Immunohistochemistry and Image Analysis: To clarify the potential biological mechanism of the key predictive factor “tumor-capsule distance”, a total of 45 PTMC patients who were diagnosed as malignant by preoperative fine-needle aspiration biopsy and positive for BRAF V600E mutation were selected. After intraoperative resection of the lesion, the surgical specimen was incised vertically along the ventral side of the thyroid gland, perpendicular to the long axis of the tumor, and the shortest distance from the tumor to the thyroid capsule was directly measured (**Figure 2B**), followed by routine pathological examination. Meanwhile, immunohistochemical staining with D2–40 was performed to evaluate lymphatic vessel distribution. Lymphatic vessel density (LVD) was assessed using the method proposed by Bono et al (23). D2-40-stained sections were scanned whole-slide using Motic EasyScanner software. Two hotspots with the densest and clearly stained lymphatic vessels were selected in the perithyroid capsule area, and the number of lymphatic vessels was counted under a 20× magnification field (corresponding to an area of 0.5 mm²), with the average value defined as “pericapsular LVD”. Similarly, two hotspots were selected in the normal thyroid tissue area far from the capsule and inside the tumor, respectively, and the average values were defined as “LVD in the area far from the capsule” and “intratumoral LVD”.

A total of 503 patients included in this study were randomly divided into a training set (n=301), a validation set (n=101), and a test set (n=101) at a ratio of 6:2:2. The training set was used for model training and hyperparameter tuning. The validation set was merged with the training set for nested cross-validation during feature selection, and subsequently served as the early stopping monitor during hyperparameter optimization. The test set remained completely independent, used solely for the final evaluation of model performance. This division strategy was intended to reduce the risk of overfitting and provide an unbiased performance estimation through an independent test set, thereby ensuring the reliability of the model on unknown data.

### 2.3 Model development and optimization

Based on the aforementioned data, we trained XGBoost and logistic regression models respectively. To enhance the reliability of feature selection, a nested cross-validation framework was adopted in this study: the training set and validation set were merged into a development set (n=402), and 5-fold stratified cross-validation was performed on this set. In each fold, the XGBoost model was trained using the training subset of the fold, and feature importance ranking was calculated based on SHAP values. Meanwhile, the predictive performance of the selected features was evaluated on the corresponding validation subset, and the area under the curve (AUC) on the independent test set (n=101) was used as the final criterion to judge the generalization ability of the feature set. The results of 5-fold cross-validation showed that the top 5 features with the average ranking appeared stably in all folds. Based on this feature set, grid search and stratified 5-fold cross-validation were employed to optimize hyperparameters on the training set (n=301), with the validation set (n=101) used as external early stopping monitoring to construct the XGBoost model. Simultaneously, a logistic regression model was established based on the above five core variables (tumor-capsule distance, microcalcification, age, BMI, and maximum tumor diameter), and the model coefficients were directly used to construct a nomogram, providing a visualized risk assessment tool. In addition, univariate and multivariate binary Logistic regression analyses were conducted in the training set to evaluate the risk factors for central lymph node metastasis (CLNM).

#### 2.3.1 XGBoost algorithm

Extreme Gradient Boosting (XGBoost) is an ensemble learning method that constructs multiple weak learners (decision trees) sequentially and combines them to form a powerful predictive model. This algorithm incorporates L1 (Lasso) and L2 (Ridge) regularization terms into the loss function, which helps prevent model overfitting and improve generalization performance.

Grid search combined with stratified 5-fold cross-validation was used to optimize the model hyperparameters on the training set, with the area under the receiver operating characteristic curve (AUC) as the optimization target. The complete hyperparameter search space and the finally selected values are as follows:

- learning_rate: search range [0.01, 0.05], finally selected 0.05. A lower learning rate combined with a larger number of iterations helps improve the robustness of the model.
- max_depth: search range (3, 5), finally selected 3. A smaller depth limits the complexity of a single tree, effectively preventing overfitting.
- gamma: search range [0.1, 0.2], finally selected 0.2. A higher gamma value makes the model more conservative in node splitting and filters out noisy splits.
- subsample: search range [0.7, 0.8], finally selected 0.7. Each tree randomly samples 70% of the training samples to increase the randomness of the model.
- colsample_bytree: search range [0.7, 0.8], finally selected 0.7. Each tree randomly selects 70% of the features for splitting.
- n_estimators: fixed at 500, and the actual number of iterations is automatically determined by the early stopping mechanism based on the performance of the validation set.
- min_child_weight: search range (1, 3), finally selected 3. A larger value can avoid the model learning local small-sample noise.
- L1 regularization coefficient (reg_alpha): search range [0.1, 0.5], finally selected 0.5.
- L2 regularization coefficient (reg_lambda): search range [0.1, 0.5], finally selected 0.5. The introduction of L1/L2 regularization further controls the model complexity.
- Positive sample weight (scale_pos_weight): Given that the positive rate of lymph node metastasis (CLNM) in this dataset is 35.55%, with a slight class imbalance, this parameter was set to 1 (i.e., no additional weighting was performed, and the model relied on the robustness of cross-validation).

To improve the interpretability of XGBoost model predictions, SHAP values were used. SHAP values can quantify the contribution of each feature to a specific prediction. Meanwhile, to explore the nonlinear and interactive effects of individual features on model predictions, SHAP dependence plots were generated.

### 2.4 Model evaluation

AUC, sensitivity, specificity and F1-score were calculated in the test set to evaluate model performance. The 95% confidence intervals were estimated via Bootstrap resampling, and the optimal cut-off value was determined by maximizing the Youden index. The receiver operating characteristic (ROC) curves, calibration curves and decision curve analysis (DCA) curves were plotted in the training, validation and test sets, respectively, to comprehensively assess the discrimination, calibration and clinical net benefit of the nomogram model. The DeLong test was applied for pairwise comparison of AUC values between different models.

External validation: A total of 101 consecutive patients enrolled from May 2023 to May 2024 in the Second Affiliated Hospital of Wannan Medical University were included as the external validation cohort, with the same enrollment period as that of the First Affiliated Hospital of Wannan Medical University, to evaluate the generalizability of the established model. Inclusion and exclusion criteria for this external cohort were strictly consistent with those for the training cohort. Collected predictive variables covered five core features adopted for model construction, including tumor capsule distance, microcalcification, age, body mass index (BMI), and maximum tumor diameter. The AUC value was calculated and the corresponding ROC curve was generated in the external validation cohort.

### 2.5 Statistical analysis

Statistical analyses were performed using R software (version 4.5.0), Python (version 3.13), SPSS (version 27.0.1), and GraphPad Prism (version 10). Model training and performance evaluation were completed in Python using the libraries pandas, NumPy, scikit-learn, XGBoost, Shap, and Matplotlib. Nomogram construction and plotting of related curves were implemented using the rms package in R software. Univariate and multivariate Logistic regression analyses were conducted with SPSS, and some statistical graphs were generated using GraphPad Prism. Descriptive statistics were expressed as mean ± standard deviation, and a P value < 0.05 was considered statistically significant.

The normality of continuous variables was verified by the Shapiro-Wilk test, and all variables did not conform to the normal distribution (p < 0.05). Therefore, the nonparametric Kruskal-Wallis H test was used to evaluate the consistency of distribution between groups. Categorical variables were analyzed by the chi-square test, and variables with low expected frequencies were analyzed by the Fisher’s exact test. The DeLong test was used to compare the AUC values between different models.

## 3 Results

### 3.1 Patient characteristics

A total of 503 patients diagnosed with PTMC were included in this study and randomly divided into training (n=301), validation (n=101), and test (n=101) sets at a ratio of 6:2:2. The baseline characteristics of the patients in these three groups are summarized in **Table 1**. No significant differences were observed among the groups in terms of age, sex, body mass index (BMI), maximum tumor diameter, history of hypertension, history of diabetes, tumor location, lymph node metastasis (LNM), Hashimoto’s thyroiditis (HT), ipsilateral nodular goiter (iNG), multifocality, tumor-capsule distance, BRAF mutation status, tumor margin, or microcalcifications. This confirmed the balanced distribution of data across the sets, thereby minimizing the potential impact of selection bias on model development.

**Table 1:** Comparison of clinical, ultrasonographic, and molecular characteristics of PTMC patients among the training, validation, and test cohorts.

Characteristic	Training Cohort (n=301)	Validation Cohort (n=101)	Test Cohort (n=101)	*P* value
Age (y)	45.37 ± 11.73	46.05 ± 11.89	44.97 ± 11.69	0.870
Sex [Male, n (%)]	71, 23.59%	23, 22.77%	18, 17.82%	0.479
BMI (kg/m^2^)	24.17 ± 3.47	24.07 ± 3.23	24.16 ± 2.90	0.893
Maximum tumor diameter (cm)	0.65 ± 0.19	0.65 ± 0.19	0.65 ± 0.21	0.961
History of hypertension [n (%)]	56, 18.60%	16, 15.84%	19, 18.81%	0.805
History of diabetes [n (%)]	13, 4.32%	6, 5.94%	4, 3.96%	0.765
Tumor location [Upper/Middle/Lower/Isthmus, n (%)]	73/139/76/13	20/46/28/7	21/47/23/10	0.481
CLNM [n (%)]	107, 35.55%	35, 34.65%	35, 34.65%	0.979
HT [n (%)]	76, 25.25%	20, 19.80%	18, 17.82%	0.226
iNG [n (%)]	15, 4.98%	2, 1.98%	6, 5.94%	0.328
Multifocality [n (%)]	36, 11.96%	17, 16.83%	16, 15.84%	0.231
Tumor-capsule distance (mm)	2.36 ± 1.41	2.25 ± 1.34	2.37 ± 1.58	0.987
BRAF mutation [n (%)]	277, 92.03%	97, 96.04%	95, 94.06%	0.356
Tumor margin [n (%)]	65, 21.59%	28, 27.72%	19, 18.81%	0.377
Microcalcifications [n (%)]	140, 46.51%	45, 44.55%	45, 44.55%	0.911

Data are presented as mean ± standard deviation or number of cases. BMI, Body Mass Index; CLNM, Central Lymph Node Metastasis; HT, Hashimoto’s Thyroiditis; iNG, Ipsilateral Nodular Goiter; PTMC, Papillary Thyroid Microcarcinoma.

The consistency of continuous variables across groups was assessed using the Kruskal–Wallis H test, while categorical variables were compared using the chi-square test. Fisher’s exact test was applied for the history of diabetes and iNG due to expected frequencies less than 5. All p-values were greater than 0.05, as detailed (in **Table 1**).

### 3.2 Feature importance and SHAP analysis

**Figure 3:**
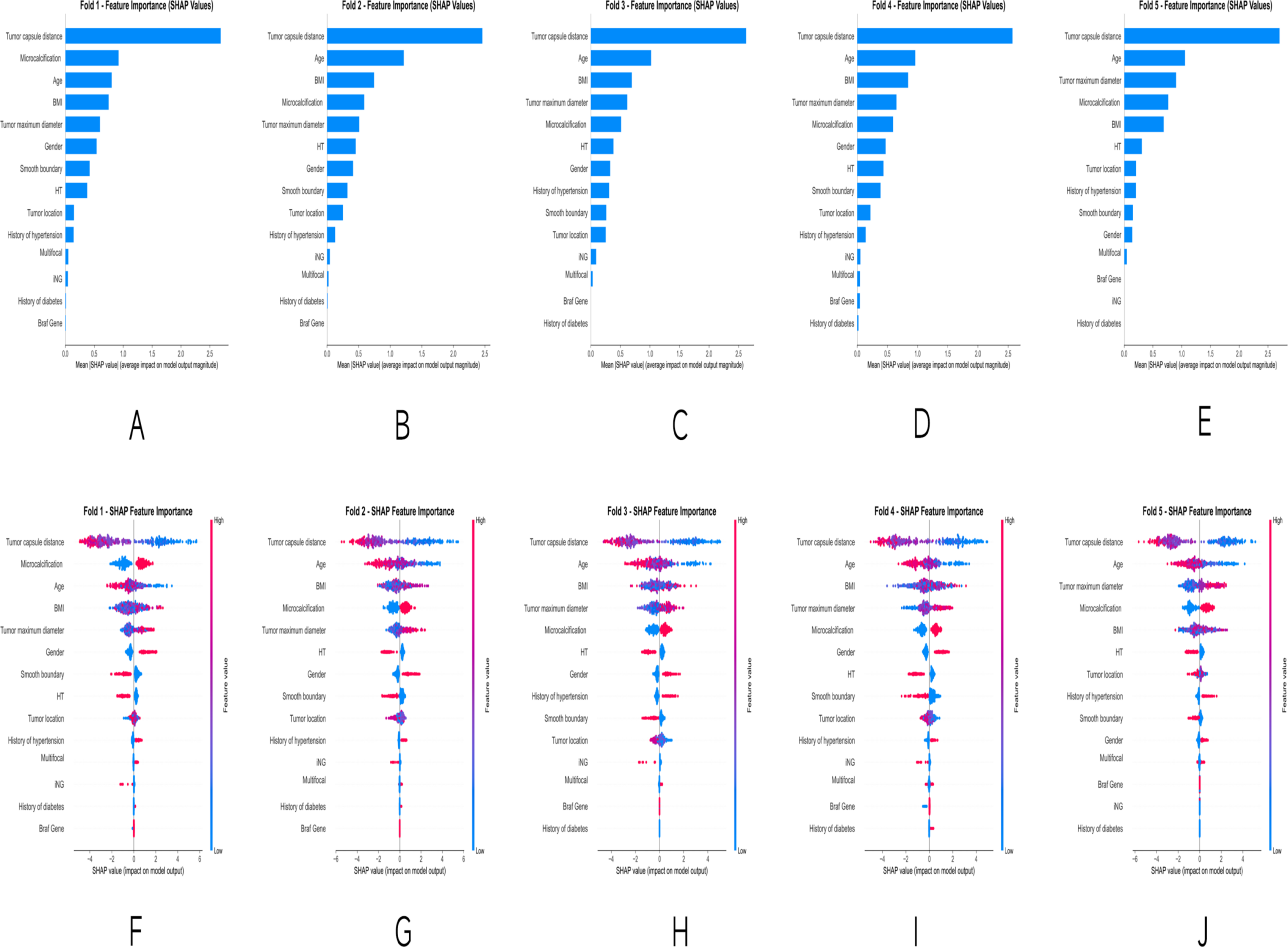
Feature importance and SHAP value distribution plots based on SHAP analysis. **(A–E)**: Bar plots of SHAP feature importance; **(F–J)**: SHAP value distribution plots. The vertical axis lists the features used in the model, while the horizontal axis represents the SHAP value, indicating the magnitude and direction of each feature’s contribution to the model output. Features are ranked from top to bottom in descending order of importance. Points distributed to the right (positive SHAP values) indicate that the feature increases the prediction, whereas points to the left (negative SHAP values) indicate a decrease in the predicted outcome. Red dots correspond to high feature values, and blue dots represent low values; the color illustrates the direction of influence of high versus low values on the prediction.

**Figure 3** presents bar charts of feature importance scores from 5-fold cross-validation based on SHAP values (**Figures 3A–E**) and SHAP value distribution plots (**Figures 3F–J**). From the bar charts of feature importance scores based on SHAP values, we found that the TOP 5 feature sets were completely consistent across the 5-fold cross-validation, with no rank changes due to different data divisions. The average AUC of the 5-fold cross-validation was 0.864 ± 0.026, and the AUC fluctuation range of each fold was 0.838 to 0.890. Based on the high consistency of the ranking of the top 5 features in the 5-fold results, this study determined the final feature set (tumor capsule distance, microcalcification, age, BMI, and maximum tumor diameter) based on the feature order of the first fold. Subsequently, an untuned model was trained on the original training cohort (n=301) using this feature set, and its AUC on the independent test cohort (n=101) was 0.884, which fell within the 95% confidence interval (0.841-0.887) of the cross-validation AUC and was basically consistent with the average performance during the cross-validation phase. This indicates that the feature selection itself is stable, and the rank fluctuation has little actual impact on the model; therefore, we finally fixed the features as follows: tumor capsule distance, microcalcification, age, BMI, and maximum tumor diameter. It is worth noting that tumor capsule distance maintained the first important rank in all cross-validation folds, showing extremely strong stability, which is highly consistent with the clinical recognition that capsule invasion is a key prognostic indicator (21). According to the SHAP value distribution plots, tumor-capsule distance, as the most important predictor of central lymph node metastasis (CLNM), was significantly negatively correlated with CLNM risk. Age was also negatively associated with CLNM. In contrast, microcalcification, maximum tumor diameter, and BMI were positively correlated with CLNM risk. Notably, there were partial red-blue overlapping regions in the SHAP value distributions of variables such as tumor-capsule distance, age, maximum tumor diameter, and BMI, suggesting that these predictors may have nonlinear relationships or threshold effects.

**Figure 4:**
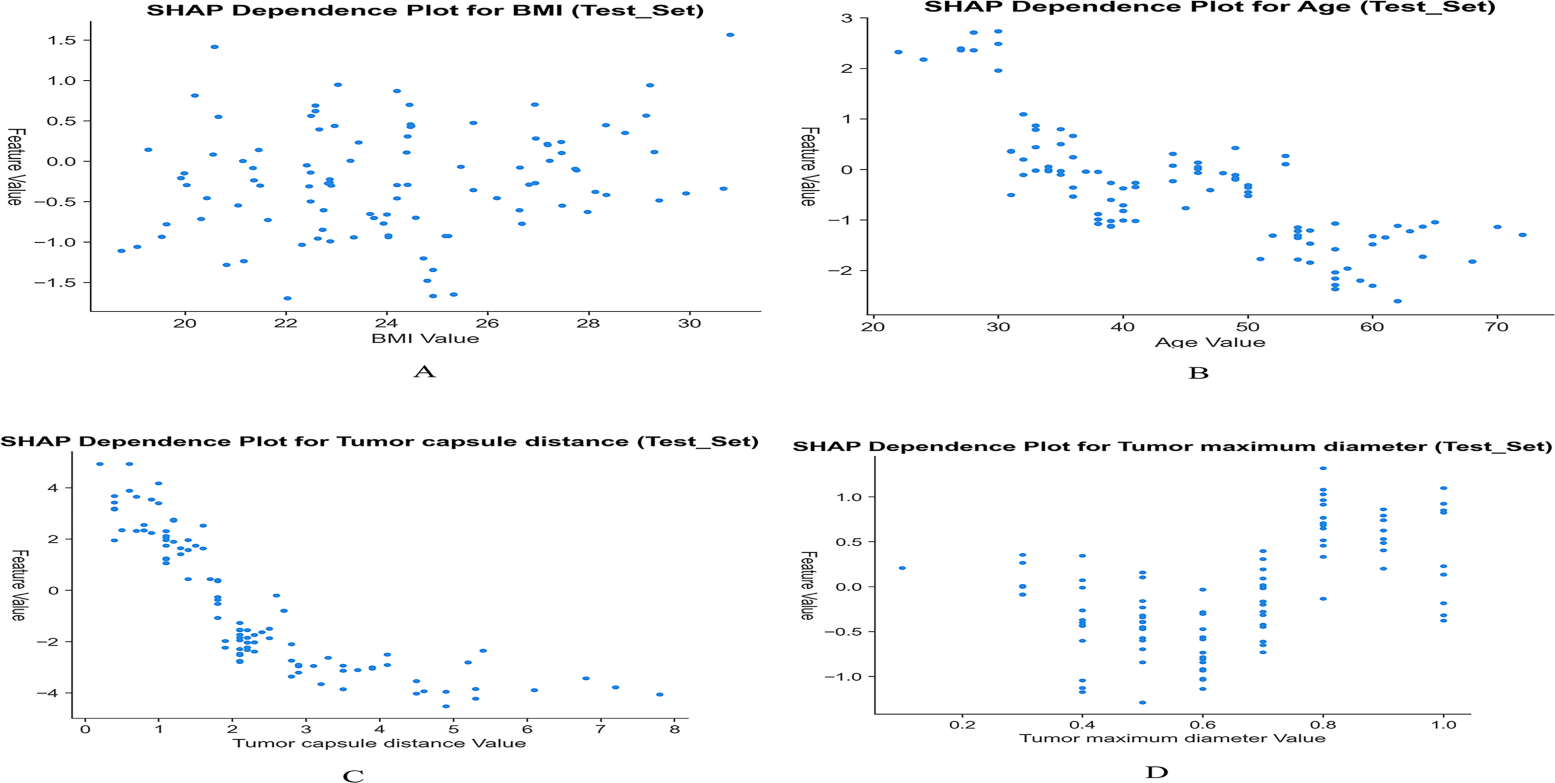
Dependence plots for each feature. Each point in the plot represents an individual patient. The y-axis shows the SHAP value, while the x-axis displays the corresponding feature value. For example, in the case of tumor-to-capsule distance, values less than 2 mm are associated with SHAP values above 0, whereas values greater than 2 mm correspond to SHAP values below 0. Thus, the threshold for predicting central lymph node metastasis (CLNM) is a tumor-to-capsule distance of 2 mm. The y-axis is scaled independently for each feature. **(A)** BMI. **(B)** Age. **(C)** Tumor capsule distance. **(D)** Tumor maximum diameter.

In **Figure 4**, each point represents a single patient. The SHAP values are displayed on the y-axis, and the corresponding feature values are displayed on the x-axis, presenting SHAP dependence plots of features with high predictive values. As shown in **Figure 4A**, the association pattern between BMI and CLNM was relatively complex: some patients with high BMI (>28) exhibited high SHAP values, but the overall data points were scattered and did not reach statistical significance. For age (**Figure 4B**), two key risk thresholds were observed at approximately 30 years and 55 years. When the tumor capsule distance was < 2 mm, the metastasis risk increased significantly, and the risk increased as the distance decreased (**Figure 4C**). It can be seen from **Figure 4D** that there was no clear monotonic relationship or significant threshold effect between the maximum tumor diameter and CLNM risk. (Due to its discrete nature, the categorical variable microcalcification was not separately plotted in the dependence plot).

The results of univariate and multivariate logistic regression analyses are recorded in **Table 2**. Univariate analysis showed that age, gender, maximum tumor diameter, Hashimoto’s thyroiditis (HT), tumor capsule distance, smooth margin, and microcalcification were significantly correlated with central lymph node metastasis (CLNM) in patients with papillary thyroid microcarcinoma (PTMC) (p < 0.05). In the multivariate analysis, these variables remained independent risk factors for lymph node metastasis (LNM) in PTMC patients (p < 0.05). Among them, age, HT, tumor capsule distance, and smooth margin were negatively correlated with CLNM in PTMC patients (p < 0.05), while male gender, maximum tumor diameter, and microcalcification were positively correlated with CLNM. Both machine learning and traditional regression analyses identified tumor capsule distance, smooth margin, microcalcification, age, HT, maximum tumor diameter, and gender as predictors of CLNM. Notably, tumor capsule distance, which ranked first in the SHAP analysis, was also a significant independent risk factor in both univariate and multivariate logistic regression analyses, indicating that it is the most stable predictor.

**Table 2:** Univariate and multivariate variable analysis of factors associated with CLNM in the training group.

	Univariate analysis				Multivariate analysis			
Factors	β	Wald	OR (95%CI)	p-value	β	Wald	OR (95%CI)	p-value
Age	-0.058	26.610	0.943 (0.923-0.964)	<0.001	-0.062	16.906	0.940 (0.913-0.968)	<0.001
Sex	-0.911	10.787	2.487 (1.444-4.285)	0.001	-0.813	4.445	2.255 (1.059-4.803)	0.035
BMI	0.034	0.955	1.034 (0.967-1.106)	0.328				
Maximum tumor diameter	2.450	12.914	11.593 (3.046-44.115)	<0.001	2.263	5.561	9.613 (1.465-63.055)	0.018
History of hypertension	-0.104	0.114	1.109 (0.608-2.024)	0.735				
History of diabetes	-0.131	0.050	1.140 (0.363-3.575)	0.823				
Tumor location (Isthmus)	ref							
Tumor location (Upper)	-0.320	0.2779	0.726 (0.221-2.382)	0.597				
Tumor location (middle)	-0.582	0.991	0.559 (0.177-1.758)	0.320				
Tumor location (lower)	-0.385	0.405	0.681 (0.208-2.228)	0.525				
HT (yes)	0.746	6.091	0.474 (0.262-0.858)	0.014	0.849	4.506	0.428 (0.195-0.937)	0.034
iNG (yes)	0.437	0.536	0.646 (0.201-2.081)	0.464				
Multiple lesions (yes)	-0.163	0.199	1.177 (0.575-2.408)	0.656				
TCD	-1.321	58.033	0.267 (0.190-0.375)	<0.001	-1.340	49.487	0.262 (0.180-0.380)	<0.001
Boundary clear (yes)	0.653	4.235	0.520 (0.279-0.969)	0.040	1.018	5.481	0.361 (0.154-0.847)	0.019
Microcalcification (yes)	-1.209	22.973	3.351 (2.044-5.495)	<0.001	-0.897	7.301	2.453 (1.279-4.702)	0.007
BRAF gene mutation (yes)	-0.795	2.358	2.215 (0.803-6.111)	0.125				

β, Regression coefficient; OR, Odds ratio; CI, Confidence interval. (All binary variables were coded as yes = 1 and no = 0 (for gender, male = 1 and female = 0). An odds ratio (OR) > 1 indicates a positive correlation (risk factor), while OR < 1 indicates a negative correlation (protective factor).).

**Figure 5:**
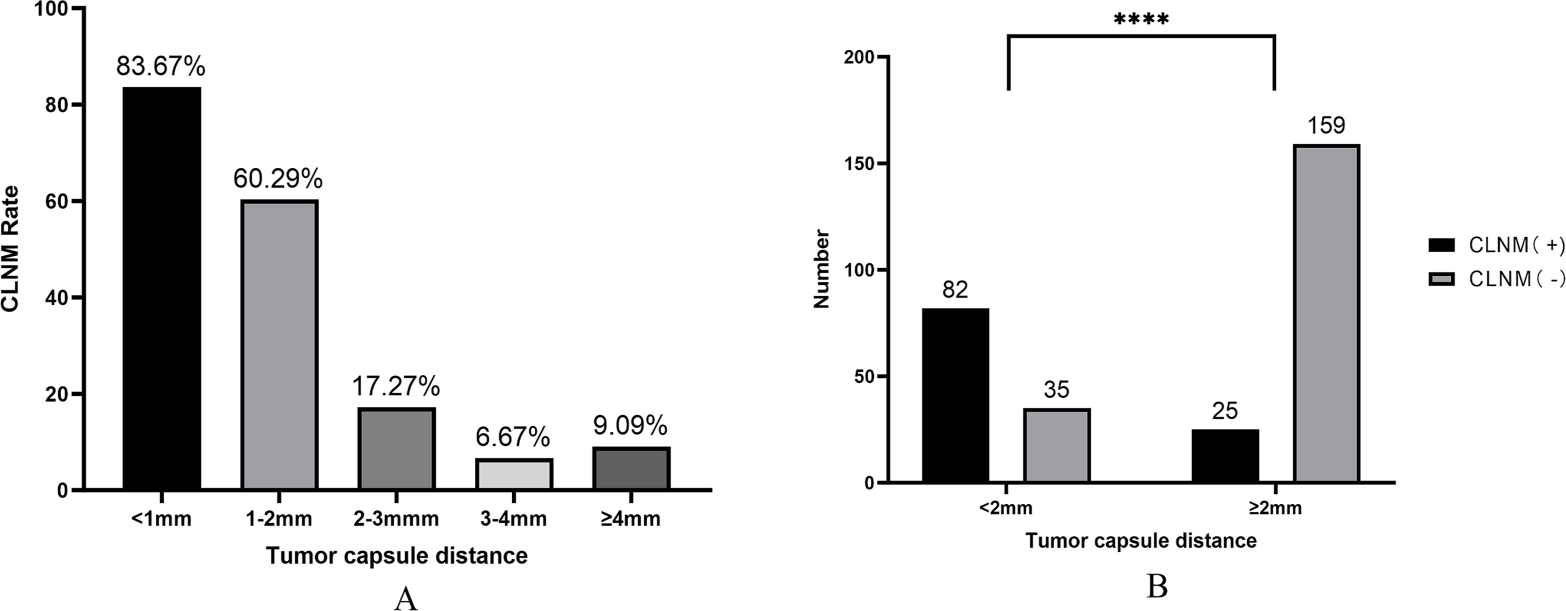
Relationship between CLNM rate and tumor-to-capsule distance. **(A)** Association between CLNM rate and tumor-capsule distance. **(B)** Correlation between CLNM and the threshold of 2 mm. **** indicates P < 0.001.

We further performed a stratified analysis to examine the correlation between tumor capsule distance and CLNM rate in PTMC patients (**Figure 5A**). When the tumor capsule distance was < 2 mm, the CLNM rate was relatively high (83.67%~60.29%). This can be explained by the presence of a rich lymphatic network around the thyroid capsule (19, 20), such that tumors close to the capsule (< 2 mm) are more likely to invade the surrounding lymphatic or blood vessels, leading to a sharp increase in metastasis risk. When the tumor capsule distance was ≥ 2 mm, the CLNM rate dropped sharply to 17.27%, and further decreased to 6.67% when the distance exceeded 3 mm. This suggests that 2 mm may be the critical distance for tumors to break through the capsule and metastasize, which is consistent with the results obtained from the aforementioned SHAP dependence plots. Further analysis confirmed that a tumor-capsule distance of < 2 mm was significantly associated with CLNM positivity (**Figure 5B**, p < 0.0001), again indicating that tumor capsule distance is a noteworthy predictor of CLNM.

### 3.3 Model performance

**Figure 6:**
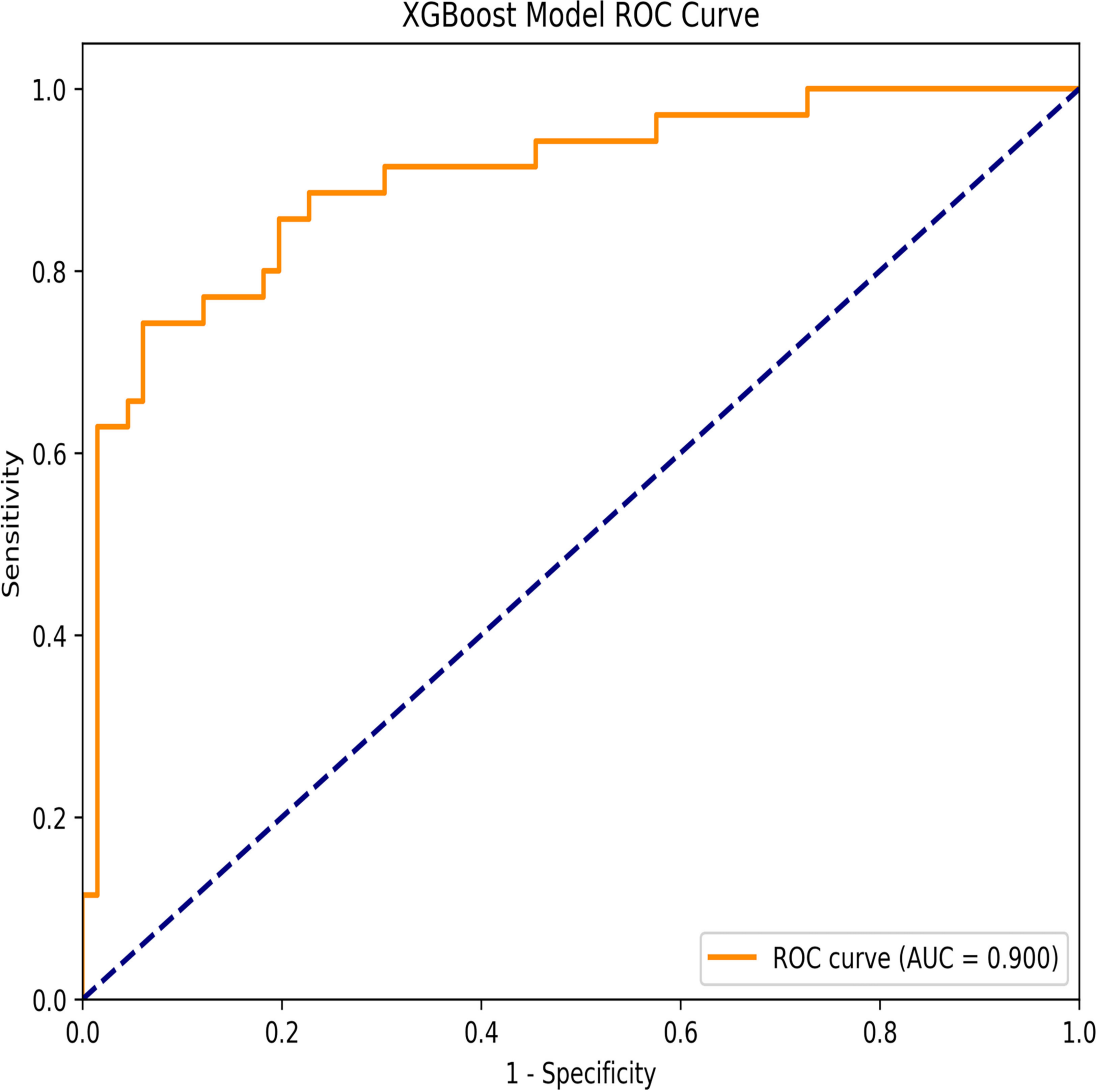
ROC curve of the XGBoost model.

The XGBoost model developed in this study demonstrated excellent predictive performance on the independent test set (**Table 3**), achieving an AUC of 0.900 (95% CI: 0.822–0.959). The ROC curve is shown in **Figure 6**, demonstrating strong discriminative ability. The optimal decision threshold was determined to be 0.32 by maximizing Youden’s index. At this threshold, the model exhibited a sensitivity of 0.857 (95% CI: 0.730–0.960) and a specificity of 0.803 (95% CI: 0.696–0.894), indicating its effectiveness in identifying true positive cases while controlling the false positive rate. The F1 score was 0.769, reflecting a good balance between precision and recall. No statistically significant difference in predictive performance was observed between the XGBoost model and the calibrated logistic regression model (DeLong’s test, P = 0.501), suggesting comparable predictive capability between the two approaches. This result indicates that, compared to the more complex machine learning model, our logistic regression model retains most of the predictive efficacy while maintaining structural simplicity, making it more suitable for the development of a clinical nomogram.

**Table 3:** Performance values of the XGBoost model.

Performance evaluation metrics	Test group data, 95% CI
AUC Sensitivity. Specificity. F1 score.	0.900 (0.8221, 0.9589) 0.857 (0.7297, 0.9630) 0.803 (0.6964, 0.8940) 0.769

### 3.4 Development and performance of the nomogram

**Figure 7:**
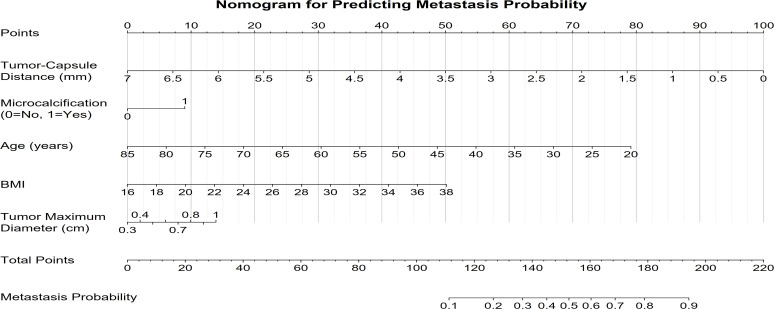
Nomogram for predicting central lymph node metastasis in papillary thyroid microcarcinoma (PTMC). This nomogram incorporates five variables: tumor-to-capsule distance, microcalcification, age, BMI, and maximum tumor diameter. Each variable is assigned a specific number of points based on its value. The total points obtained from all variables are then converted into a predicted probability of lymph node metastasis.

**Figure 8:**
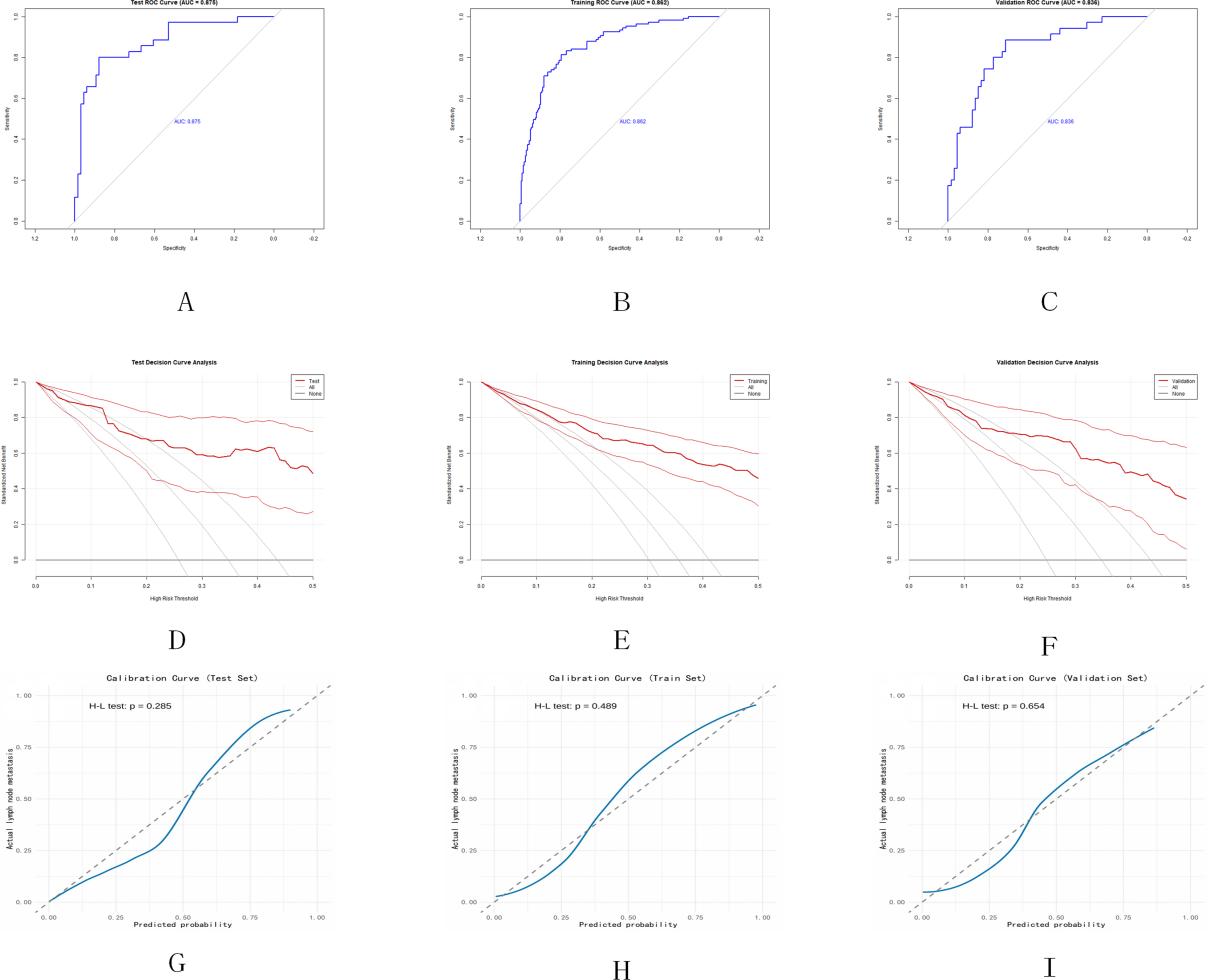
Performance of the nomogram for predicting central lymph node metastasis in papillary thyroid microcarcinoma. **(A–C)**: ROC curves of the nomogram in the training, validation, and test sets, with AUC values of 0.862, 0.836, and 0.875, respectively. Panel **(D–F)** Calibration curves of the nomogram, showing good alignment with the reference line in all three subsets, Panel **(G–I)** Decision curve analysis of the nomogram. Across all groups, the model demonstrated higher net benefit than both null strategies within a threshold probability range of 10% to 50%.

Based on the logistic regression model, we developed a nomogram to predict the probability of CLNM in PTMC patients, as shown in **Figure 7**. This model incorporated five variables, namely tumor capsule distance, microcalcification, age, BMI, and maximum tumor diameter. The regression coefficients of each variable are detailed in **Table 4**. Each variable was assigned a score according to its regression coefficient, and the total score was calculated by summing these individual scores. Subsequently, this total score was converted into a predicted probability of lymph node metastasis. **Figure 8** describes the functional evaluation of the nomogram model. ROC analysis showed that the model had significant discriminative efficacy, with AUC values of 0.862 (95% CI: 0.823-0.905), 0.836 (95% CI: 0.748-0.914), and 0.875 (95% CI: 0.811-0.946) in the training, validation, and test cohorts, respectively. The calibration curves showed a high consistency between the predicted probabilities and the actual observed values, indicating good calibration of the model. To further clarify the clinical applicability of the model, we evaluated its performance at different decision thresholds: when the threshold was set at 0.55, the model exhibited a high specificity of 0.95, meaning it could identify low-risk patients without CLNM with extremely high reliability (95%), but its sensitivity was 0.60. When the threshold was set at 0.70, the model achieved a sensitivity of 0.88, which could effectively capture the vast majority (88%) of patients with lymph node metastasis, while the specificity remained at 0.82. Decision curve analysis further confirmed its clinical value: within a wide range of probability thresholds, the net benefit of the model was superior to the strategies of “treating all patients” or “treating no patients”.

**Table 4:** Line chart model: table of logistic regression coefficients.

Factors	β
intercept	0.4439665551208358
TCD	-0.7567364517314953
Microcalcifications	0.4774820548200755
Age	-0.06450609558546333
BMI	0.12071274195172972
Maximum tumor diameter	1.0526881505949754

Microcalcification is a binary variable (yes=1, no=0)

### 3.5 External validation

**Figure 9:**
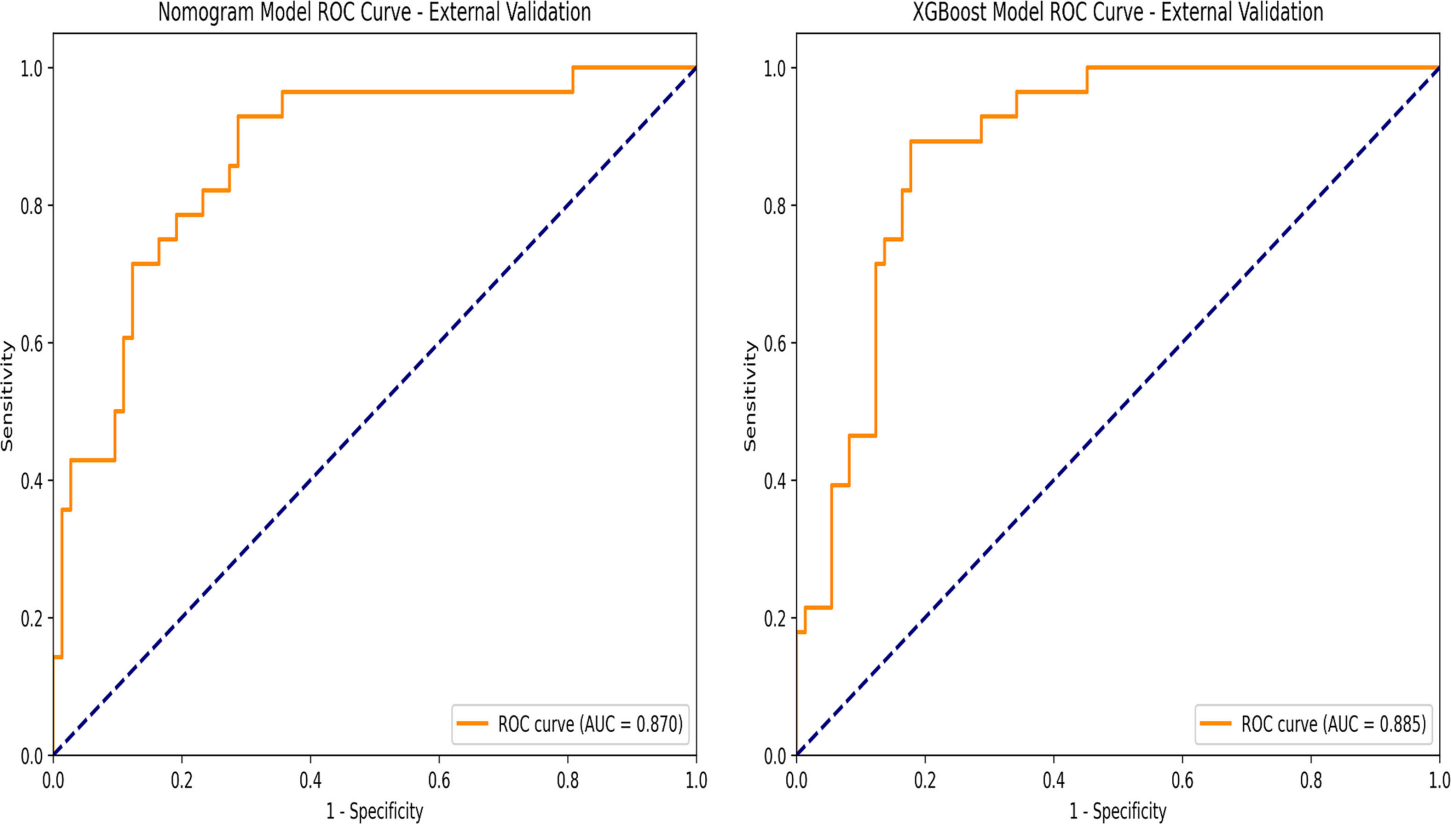
ROC curves of the XGBoost model versus the nomogram in the external validation cohort.

An independent external validation cohort comprising 101 cases was utilized to evaluate the model’s performance. The baseline characteristics of this cohort are detailed in **Table 5**. Both the XGBoost model and the nomogram model demonstrated excellent discriminative ability, with areas under the receiver operating characteristic curve (AUC) of 0.885 and 0.870, respectively (DeLong’s test, p > 0.05). The comparable performance of both models in external validation confirms the robust predictive performance of the developed nomogram. The ROC curves for both models are presented in **Figure 9**.

**Table 5:** Basic information of the external center verification team.

Factors	Mean ± standard deviation or number of cases.
TCD (mm)	2.24 ± 1.06
Microcalcifications (%)	53, 52.48%
Age (y)	47.15 ± 11.70
BMI (kg\m^2^)	24.64 ± 3.55
Maximum tumor diameter (cm)	0.63 ± 0.24
CLNM (%)	28, 27.72%

### 3.6 D2–40 immunohistochemistry for lymphatic vessel staining

**Figure 10:**
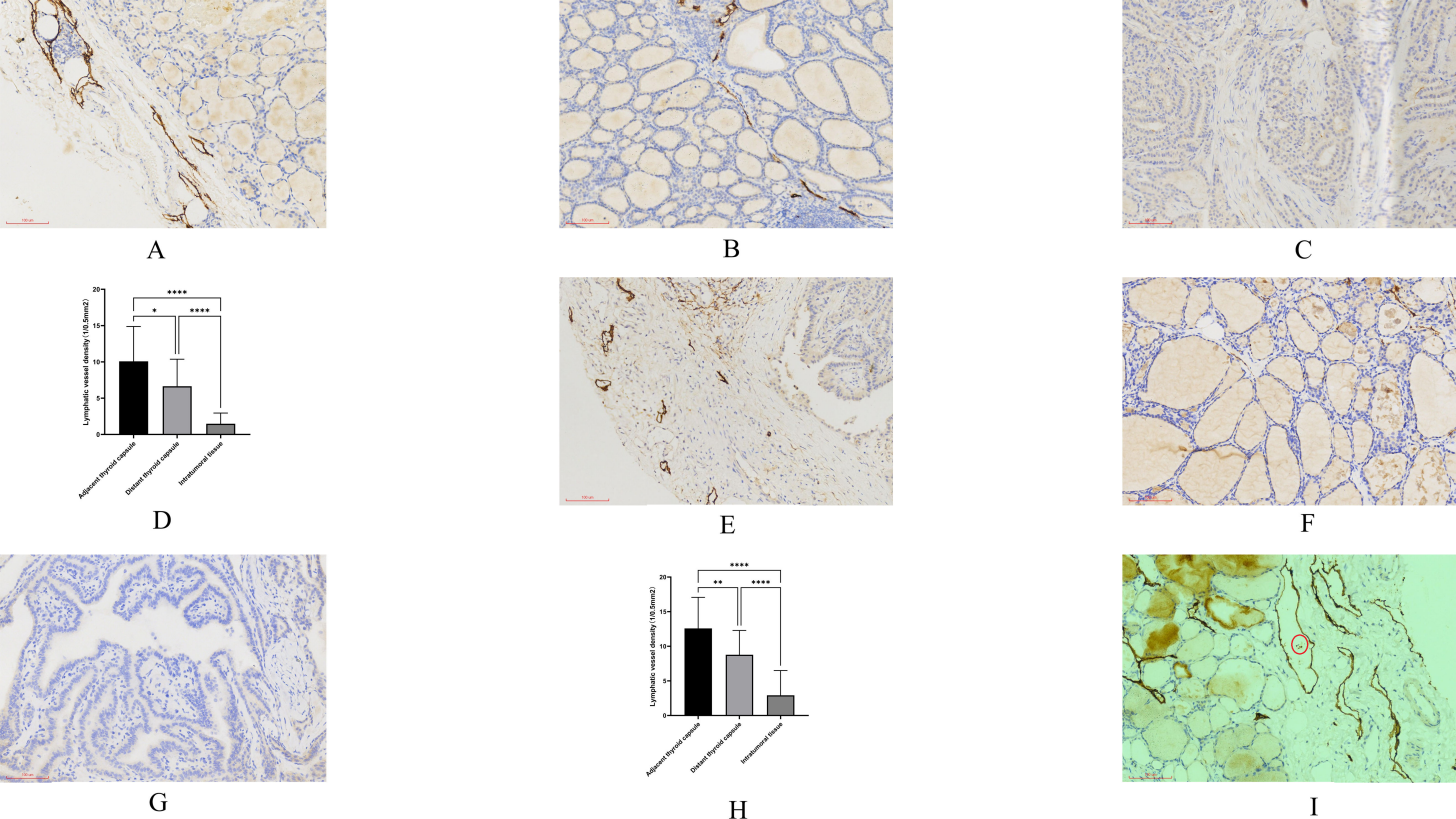
D2–40 immunohistochemical staining. **(A)** Pericapsular thyroid tissue; **(B)** Thyroid tissue in the distant non-tumorous capsular region; **(C)** Intratumoral tissue; **(D)** Comparison of lymphatic vessel density among different regions in the CLNM-positive group; **(E)** Pericapsular thyroid tissue; **(F)** Thyroid tissue in the distant non-tumorous capsular region; **(G)** Intratumoral tissue; **(H)** Comparison of lymphatic vessel density among different regions in the CLNM-negative group; **(I)** Cancer cells are observed in the lymphatic vessels near the capsule.

According to D2–40 immunohistochemical staining (**Figure 10**), lymphatic vessel density was highest in the capsular region of the thyroid (**Figures 10A, E**), followed by non-neoplastic thyroid tissue away from the capsule (**Figures 10B, F**). In contrast, lymphatic vessels were sparse within the tumor (**Figures 10C, G**), and were particularly rare or showed unclear lumen structures in the central tumor area.

A total of 45 patients with postoperatively confirmed papillary thyroid microcarcinoma (PTMC) were included in this study. Intergroup comparisons revealed that in the lymph node metastasis (LNM) group (**Figures 10A–C**), intratumoral lymphatic vessel density (LVD) was significantly lower than that in normal thyroid tissue (p < 0.001). Additionally, LVD in normal thyroid tissue distant from the capsule was also reduced compared to that in the pericapsular area (p < 0.05; **Figure 10D**).

In the group without lymph node metastasis (**Figures 10E–G**), intratumoral LVD was similarly significantly lower than that in normal thyroid tissue (p < 0.001). Moreover, LVD in areas away from the capsule was decreased compared to the capsular adjacent region (p < 0.01; **Figure 10H**).Simultaneously, in some cases, it was observed that the lymphatic vessels near the thyroid capsule were dilated, and tumor cells with large, deeply stained nuclei were seen (**Figure 10I**).

## 4 Discussion

Controversy remains regarding the clinical management strategy for low-risk papillary thyroid microcarcinoma (PTMC). Based on data from 604 PTMC patients across two medical centers, this study found that the incidence of central lymph node metastasis (CLNM) was 33.9%, which is consistent with the results reported in multiple previous studies (27%–65%) (24–26). Feature selection was performed using the XGBoost algorithm, and five key predictive variables were finally identified in this study for constructing a CLNM prediction nomogram, including maximum tumor diameter, microcalcification, BMI, age, and tumor-to-capsule distance. Univariate and multivariate Logistic regression analyses further showed that all variables except BMI were independent risk factors for CLNM (P < 0.05).

Zhang et al. (27), based on a study of 337 patients with papillary thyroid carcinoma (PTC), pointed out that the maximum nodule diameter is an important predictor of lymph node metastasis, which may reflect increased tumor invasiveness. However, the SHAP dependence analysis in this study did not find a significant threshold effect between this indicator and central lymph node metastasis (CLNM), and this difference may be related to the relatively indolent biological characteristics of PTMC. As a typical ultrasonic feature of PTC, microcalcification (diameter < 2 mm) has been confirmed to be associated with LNM in multiple studies (28, 29), and its mechanism may involve tumor microthrombus formation or extracellular glycoprotein deposition; the hyperechoic foci appearing in metastatic lymph nodes are also considered to be related to thyroglobulin accumulation (30, 31). Existing studies have confirmed that insulin resistance and metabolic syndrome are positively correlated with thyroid cancer (32). Papillary thyroid carcinoma (PTC) patients complicated with hypertension, especially males over 40 years old, are prone to present aggressive clinicopathological characteristics (33). However, the univariate and multivariate analyses revealed that hypertension and diabetes were not significantly associated with central lymph node metastasis (CLNM) in this study (all P > 0.05).Logistic regression showed no significant independent association between BMI and CLNM, but SHAP analysis suggested that there may be a nonlinear or conditional effect. This finding is consistent with the results of Ding et al. (34): although elevated BMI does not directly increase the risk of CLNM, a significant increase in the number and proportion of metastatic lymph nodes can be observed in the high BMI group, indicating its indirect impact on metastatic behavior. In addition, age was negatively correlated with LNM, and risk turning points were identified at approximately 30 and 55 years old, which is consistent with multiple previous studies (29, 35). The underlying mechanisms may be related to age-related hormonal changes (36), tumor proliferative activity (37), and accumulation of specific gene mutations (38).

Currently, studies on the application of nomograms to predict central lymph node metastasis (CLNM) in patients with papillary thyroid microcarcinoma (PTMC) remain relatively limited (39). In this retrospective study, we constructed and validated a nomogram model based on machine learning methods to predict the CLNM risk in PTMC patients in a personalized and quantitative manner using the above five risk factors. Compared with previous studies, this model has the following advantages: (1) Relying on a robust feature selection process, the model construction basis is more reliable; (2) While maintaining excellent discriminative performance (AUC > 0.85), it exhibits good probability calibration ability; (3) Presented in the form of a nomogram, it has excellent intuitiveness and ease of use, which greatly promotes clinical transformation and application, and effectively solves the “last mile” problem in the practical application of complex artificial intelligence models. In terms of feature selection, this study differs from the strategy of Lapa et al. (40): instead of including all variables, it selected five stable and important core predictive factors through XGBoost algorithm and cross-validation, thereby enhancing the robustness and generalizability of the model and effectively avoiding overfitting. Compared with traditional nomogram models such as those developed by Yang (39) and Wang (41)(with AUC of 0.69 and 0.711, respectively), adopted advanced machine learning-based feature screening strategies to ensure that the enrolled predictors possessed both satisfactory predictive efficacy and robust stability. The final constructed nomogram model achieved a notably higher discriminative performance with an AUC of 0.875. Moreover, the model maintained favorable and stable predictive capacity in the independent external validation cohort (AUC = 0.870), preliminarily demonstrating good generalizability across different medical centers. Calibration curves showed good consistency between the actual incidence of CLNM and the predicted probability. Decision curve analysis results indicated that within a wide range of threshold probabilities, the clinical net benefit of this nomogram model was superior to the strategies of “treating all patients” or “treating no patients”. Although similar to the XGBoost model developed by Lapa et al., the model in this study also showed superior discriminative performance (AUC > 0.85), it is presented in the form of a nomogram, which overcomes the limitation that complex machine learning models are often regarded as “black boxes”. It can achieve real-time risk assessment without software assistance, and has certain clinical practical value. According to our results, when the model’s predicted probability is higher than 0.70, its sensitivity in identifying high-risk populations for CLNM is about 88% and specificity is about 82%, suggesting that the model has a certain ability to distinguish high-risk and low-risk patients at this threshold. Therefore, this scoring result may provide a reference for preoperative risk stratification, assist clinicians in more adequate communication and shared decision-making with patients, for example, when weighing whether to perform simultaneous central lymph node dissection. When the predicted probability is lower than 0.55, the model has high specificity (about 95%) and good credibility of negative results, which helps to screen individuals with relatively low metastasis risk. However, regardless of the prediction interval, the output of the model is only one of the decision reference information, and the final diagnosis and treatment plan still needs to be comprehensively judged based on various factors such as ultrasound, pathology, and patient wishes. In conclusion, the nomogram model constructed in this study has good discriminative ability, calibration performance, and clinical interpretability in predicting the CLNM risk in PTMC patients.

Previous studies have shown that thyroid capsule invasion is a strong predictor of LNM in PTMC. However, most PTMCs rarely invade the outer capsule, while some PTMCs present with lymph node metastasis (42). We included the ultrasound-measured tumor-capsule distance as a continuous variable in the analysis and observed a certain association between this indicator and the CLNM rate in PTMC patients. Further analysis through SHAP dependence plots and grouped descriptive statistics (**Figure 5**) revealed that the proportion of CLNM increased significantly when the distance was less than 2 mm, and decreased significantly when the distance was greater than 2 mm. This phenomenon suggests that 2 mm may be a potentially referenceable cutoff value, but its exact application value needs to be further confirmed by future studies. D2–40 is a commercially available monoclonal antibody against human Podoplanin, a transmembrane mucosal protein expressed in lymphatic endothelial cells, which has been described in various lymphatic tumors including lymphangioma, Kaposi’s sarcoma, hemangioendothelioma, and non-vascular tumors (43). In differentiated thyroid carcinoma, D2–40 can display the distribution of lymphatic vessels in the gland (44). We used D2–40 to label lymphatic endothelial cells (45), and the results showed that regardless of lymph node metastasis, the lymphatic vessel density (LVD) in thyroid tissue near the capsule was significantly higher than that in areas far from the capsule and inside the tumor, which is consistent with the view described by Di Tommaso that the perithyroid capsule is rich in lymphatic networks (19). A study by Pereira et al. pointed out that lymph node metastasis of PTC is not related to intratumoral LVD, but depends on whether tumor cells invade the rich lymphatic network in the surrounding normal thyroid tissue (46). This study found that lymphatic vessels near the capsule are more dense, and in some cases, dilated peritumor-capsule lymphatic vessels with cancer cells were observed (**Figure 10I**), indicating that this area is more prone to lymphovascular invasion (LVI), thereby increasing the risk of lymph node metastasis (LNM). This finding may partially explain the association between tumor-capsule distance and CLNM rate observed in this study, which is also consistent with the results of multiple solid tumor studies that regard LVI as a predictor of lymph node metastasis and prognosis (47–49).

However, this study still has limitations. First, the study adopted a single-center retrospective design, and the included population were all patients who underwent surgery, excluding cases under active surveillance. This means that the model was developed in a surgically selected population, and its predictive performance in a broader PTMC population, especially in low-risk patients under active surveillance, remains unclear, with the possibility of spectrum bias. Second, although the external validation cohort was from an independent hospital, it belonged to the same regional medical system as the modeling cohort and had the same time period, which essentially reflects the stability within the region; further verification in prospective cohorts from different regions and hospitals of different levels is needed in the future. Third, the 2 mm cutoff for the tumor-capsule distance was observed through SHAP analysis and grouped descriptive statistics, which is an exploratory finding, and the reliability of its use as a clinical cutoff value needs to be further confirmed by prospective studies. Finally, D2–40 immunohistochemical analysis included only 45 PTMC patients who were diagnosed by preoperative FNA and positive for BRAF V600E mutation; the small sample size and certain molecular subtype selection bias mean that it can only be regarded as an exploratory pathological clue, and further verification with an expanded sample size is needed in the future. In the future, multi-center prospective studies including active surveillance cohorts should be carried out to further verify the clinical decision-making auxiliary value of the model.

## 5 Conclusion

This study successfully constructed and validated a nomogram model based on XGBoost and Logistic regression for predicting the risk of central lymph node metastasis (CLNM) in patients with papillary thyroid microcarcinoma (PTMC). In addition to developing a practical clinical prediction tool, this study also found through pathological analysis that the “tumor-thyroid capsule distance”, a key predictive factor, is closely associated with the increased density of lymphatic vessels near the capsule, which provides a preliminary anatomical explanation for the predictive ability of the model. In summary, this nomogram can be used as an auxiliary tool for assessing the risk of CLNM in PTMC patients, providing a scientific reference for individualized clinical decision-making.

## Data Availability

The original contributions presented in the study are included in the article/supplementary material. Further inquiries can be directed to the corresponding author.
